# Improving the Quality of Antenatal Care Using Mobile Health in Madagascar: Five-Year Cross-Sectional Study

**DOI:** 10.2196/18543

**Published:** 2020-07-08

**Authors:** Anne Caroline Benski, Nicole C Schmidt, Manuela Viviano, Giovanna Stancanelli, Adelia Soaroby, Michael R Reich

**Affiliations:** 1 Takemi Program in International Health Harvard TH Chan School of Public Health Boston, MA United States; 2 Katholische Stiftungshochschule München University of Applied Science Munich Germany; 3 Ospedale Regionale di Lugano Ente Ospedaliero Cantonale Lugano Italy; 4 Vita-Salute San Raffaele University Milano Italy; 5 Centre Médico-Chirurgical Saint Damien Ambanja Madagascar; 6 Department of Global Health & Population Harvard TH Chan School of Public Health Boston, MA United States

**Keywords:** mobile health, maternal health, antenatal care, quality of care, mobile phone

## Abstract

**Background:**

Despite many efforts, maternal mortality remains a major burden in most developing countries. Mobile health (mHealth) has the potential to improve access to obstetric care through apps that help patients and providers.

**Objective:**

This study aimed to use mHealth to provide antenatal care (ANC) to 1446 pregnant women in a rural area in Madagascar and evaluate the quality of ANC provided by an mHealth system designed to change the behaviors of providers and patients.

**Methods:**

We included 1446 women who attended ANC visits in rural Madagascar from 2015 to 2019 using an mHealth system called Pregnancy and Newborn Diagnostic Assessment (PANDA). This cross-sectional study used data from different participants, with information collected over several years, to analyze the outputs related to the quality of ANC over time. Specifically, we examined the timing of the first ANC visit, the relationship between the visit duration and the risk factors among pregnant women, and the number of ANC visits per woman.

**Results:**

Following the implementation of the mHealth system in 2015, we observed that women started to come earlier for their first ANC visit; more women attended their first ANC visit in the second trimester of pregnancy in 2019 than in the previous years (*P*<.001). In 2019, fewer women attended their first ANC visit in the third trimester (57/277, 20.6%) than in 2015 (147/343, 42.9%). There were statistically significant associations between the ANC visit durations and the risk factors, including age (>35 years; 25.0 min, 95% CI 24.0-25.9), educational level (longer visit for women with lower than primary education and for women who attended university and shorter for women with primary school–level education; 40.7 min, 95% CI 30.2-51.3 and 25.3 min, 95% CI 24.4-26.3 vs 23.3 min, 95% CI 22.9-23.8; *P*=.001), experience of domestic violence during pregnancy, gravidity, parity, infectious diseases (HIV, malaria, and syphilis), and level of anemia. Statistically significant associations were observed for all quality indicator variables. We observed a statistically significant increase in the number of ANC visits per woman over time from 2015 to 2017; the number of ANC visits per woman then became stable after the third year of implementing the PANDA mHealth system.

**Conclusions:**

This study shows the potential of an mHealth system to improve the quality of ANC, change provider behavior by standardizing ANC visits, and change patient behavior by increasing the willingness to return for subsequent visits and encouraging ANC attendance early in pregnancy. As this is an exploratory study, further studies are necessary to better understand how mHealth can change behavior and identify the conditions required for behavioral changes to persist over time.

## Introduction

### Background

Mobile health (mHealth) tools are an innovative technology that can allow patients and their health care providers to effectively access medical data before, during, and after medical appointments. mHealth has the potential to improve the quality of health care through apps that can facilitate communication between patients and health care providers [1]. In the past few decades, most health-related apps have been created for patients rather than for providers [2,3].

Maternal mortality has decreased dramatically in low- and middle-income countries (LMICs), declining by 45% from 2008 to 2013 [4]; however, around the world, as many as 800 women continue to die each day because of complications from pregnancy and delivery [5]. Up to 99% of maternal deaths worldwide occur in LMICs, and researchers have postulated that a lack of access to high-quality maternal and newborn care is a major reason for maternal deaths in these countries [6,7]. Antenatal care (ANC) provides a unique opportunity for screening, diagnosis, and health promotion among pregnant women and their families and communities [8]. Appropriate utilization of ANC services is associated with improved maternal and newborn health as well as a reduction in maternal deaths during pregnancy and childbirth [9-11]. Due to the benefits of ANC, the World Health Organization (WHO) recommends that pregnant women should attend at least four ANC visits to increase opportunities for risk identification, management of pregnancy and/or comorbidities, and health promotion, and since 2016, the WHO recommends eight contacts with health care providers during pregnancy [12]. Delays in the recognition and management of clinical problems that may arise during pregnancy can increase maternal and neonatal mortality [13].

Expanded use of mHealth could help improve health care quality in the context of LMICs [14]. mHealth can offer technical support to assist health care providers in data collection and retrieval, and, even more importantly, it can support the clinical practice of health care providers and improve the quality of care and interactions between patients and health care workers [15]. If mHealth is capable of increasing patients’ satisfaction with the clinical approach, this higher satisfaction may translate into increased attendance at clinical visits, which may ultimately reduce adverse obstetric and neonatal outcomes. However, current evidence on this topic is scarce, and little is known about how mHealth can influence the clinical practice of health care providers and the quality of services [16,17].

### Objectives

We conducted a study in Madagascar using an mHealth system to record and access women’s data during their ANC visits. The main goal of this study was to evaluate the quality of ANC provided with mHealth by measuring the adherence to ANC visits, the timing of the first ANC visit, and the duration of the visits.

## Methods

### Study Setting and Collaboration

This cross-sectional, observational study was conducted from January 2015 to September 2019 in the Ambanja district in northwestern Madagascar. Data related to ANC visits were collected using an mHealth system called Pregnancy and Newborn Diagnostic Assessment (PANDA). The local health authorities in Ambanja, Madagascar, and the Human Research Ethical Cantonal Board of Geneva, Switzerland (Comité d’éthique de la recherche CER 14-217; project number: CCER PB_2017-00641) approved the study.

We implemented an mHealth system to support providers in conducting ANC visits in Madagascar. Maternal mortality in Madagascar has decreased by over 50% in the last 20 years—from 776 to 353 deaths per 100,000 live births—but maternal mortality remains high and the Millennium Development Goal 5 has not been reached [18]. In the northern part of the country, 82.1% of women receive at least one ANC visit, but only 44% receive four or more ANC visits [19]. In the Ambanja district, only 58% of pregnant women attend four or more ANC visits with any kind of health care provider [20].

The Ambanja district is a rural area with a population of 240,000 inhabitants [21]. Farming is the main economic activity in the region. This study was conducted in the 2 main hospitals in Ambanja city: the Centre Médico Chirurgical Saint Damien (a private nonprofit organization) and Centre Hospitalier de Référence du District de Ambanja (the district public hospital). In the city, ANC is provided in these 2 hospitals and in 18 dispensaries within a 200-km radius of the Ambanja district. In 2015, we provided ANC with the PANDA system in both urban and rural areas; however, in 2016, we changed the strategy and excluded rural dispensaries, providing ANC exclusively in the city of Ambanja to allow for proper follow-up.

A collaboration between the University Hospitals of Geneva and the Centre Médico Chirurgical was established in 2010 for a cervical cancer program and was continued in 2015 with the implementation of the PANDA system. The PANDA team collaborates with the Ministry of Health of Madagascar.

### The PANDA System Intervention

The PANDA mHealth system was first implemented in 2015 in a pilot study to assess the system’s feasibility and usability in Madagascar [22]. The program was designed to support health care providers who received training on using a smartphone app and on conducting a standardized ANC visit. This mHealth system encourages providers to perform a standardized ANC visit as the program requires the provider to go through all the sections of a standard ANC visit, without skipping any section from the beginning to the end. Standardization is an intended result of using an mHealth system such as PANDA. PANDA is a mobile app that facilitates the collection of information during ANC and postpartum care visits according to WHO recommendations. The collected data are automatically transmitted to the medical unit’s web-based database, which stores a digitized medical record for each patient.

The system comprises the following 3 elements. First, *PANDA point of care* is a kit containing all the instruments needed to take measurements such as height, weight, body temperature, and blood pressure and to screen for syphilis, HIV, malaria, anemia, gestational diabetes, urinary tract infections, and malnutrition. Second, the *PANDA app* is an Android smartphone app made up of 5 sections: sociodemographic information, medical and obstetric history, clinical screening test results, health education and birth plan, and postpartum care. It is an app mostly based on icons and illustrations with very little text. A quarter of each visit is dedicated to educating the patient regarding warning signs during pregnancy, labor, and postpartum as well as birth preparedness. The education section of the visits also focuses on breastfeeding recommendations and overall well-being of women. Alert functions are integrated into the PANDA app to notify the provider of abnormal clinical results, technical problems, or missing patient information, thereby assuring a complete assessment [23]. The providers must go through all 4 modules; otherwise, it is not possible to close the visit. Third, the *PANDA medical unit* is a web-based database that captures the data and results collected during the ANC and postpartum visits and enables the hospital team to open a computerized medical record for each woman to make a diagnosis and define the frequency of follow-up. In addition, after all the data were collected for this study, the PANDA medical unit allowed the team to stratify pregnancies by area, time, and risk conditions. The on-site medical team has access to the PANDA database via username and password; all data are encrypted to ensure privacy. Patients were informed, and they agreed to their data being transmitted to and saved in the medical unit to create their medical records. Patients were asked to provide both paper and electronic consent.

Data were collected during the women’s first and subsequent ANC visits. The 3 following types of information were collected: (1) sociodemographic characteristics; (2) medical, surgical, and obstetric history; and (3) results from screening to detect obesity or malnutrition, hypertension or preeclampsia, anemia, HIV, syphilis, malaria, diabetes, infections, and other conditions. The PANDA system collected data on at least 75 items per woman on their past and current medical and obstetric history as well as clinical screening data.

Since 2015, we have trained 13 providers to use the PANDA system. In the PANDA medical unit, it is possible to track the content of the visits, including each provider’s activities, such as the number of visits conducted and eventual errors in completing the visits, thus allowing the team to build a learning curve for each provider.

### Sample Description

A total of 1446 pregnant women fulfilled the inclusion criteria and were enrolled in the study. All pregnant women, regardless of age or stage of pregnancy, were eligible to participate in the study. The only exclusion criterion was the inability to understand or act as described in a previous publication explaining the acceptability and feasibility of the PANDA mHealth device [22]. As ANC with the PANDA mHealth system was offered to all women receiving routine ANC, the sample is considered to be representative.

### Statistical Analysis

We used a convenience sample of 1446 women. We planned to recruit patients from 2015 until the end of September 2020, which resulted in a total of 1446 women.

The data collected with PANDA were digitized as electronic medical records, which were used to analyze maternal morbidity and evaluate the quality of the ANC. Continuous variables are presented as mean (SD) or median, and categorical variables are presented as frequencies and relative percentages. The proportions of patients who tested positive for syphilis, HIV, or malaria are provided with their 95% CIs. Comparisons of categorical variables by year were performed using the chi-square test, and the mean durations of ANC visits were compared by the different categorical variables using the nonparametric Kruskal-Wallis test. We assessed the associations of different patient characteristics, place of residence, year, and visit order (independent variables) with visit duration (dependent variable)—first at the univariate level, using mixed linear regression models with the patient as a random factor to take into account repeated measurements within patients. We then constructed a parsimonious multivariable mixed linear regression model including all the variables that were significantly associated with the visit duration at *P*=.04. We reported the estimated marginal mean duration and 95% CI for each independent variable from the multivariable model and the associated *P* values for each category of the variables. Stata Statistical Software, release 16 (StataCorp), was used to describe the study population and to analyze the indicators of the ANC quality.

## Results

### Demographic Information of the Participants

Most of the 1446 women in the study were recruited in the city of Ambanja. The first table in Multimedia Appendix 1 summarizes the participants’ demographic and obstetric history. The mean age of the women was 24.4 years (SD 6.8), and the majority (1185/1441, 82.23%) of the participants were married or living with a partner. Most of the women had primary school–level education or lower, and most (1337/1441, 92.78%) of the participants did not have running water at home. Multiparas accounted for 60% of the participants. In our sample, more than one-third of the pregnancies were unplanned. Data on unplanned pregnancies were only included in the system in 2018 as we noticed during the visits that the women very often said that their pregnancies were not planned. In addition, data on contraceptive use before pregnancy were added in 2018 and 2019; of the women who reported previous contraceptive use in these years, 51.7% (170/329) used long-acting progesterone methods and 32.2% (106/329) used traditional methods. Only 154/985, 15.63% of the participants had been vaccinated for tetanus. A total of 4.66% (67/1438) of women reported experiencing domestic violence during their pregnancy; importantly, several women only reported this at the end of their ANC visits, after the education session on violence.

Table 1 summarizes the status of syphilis, HIV, and malaria among the pregnant women included in this study. HIV prevalence was 1.3% (95% CI 0.7-1.96%). The prevalence of syphilis infection in our sample was 2.8% (95% CI 2.0-3.8%).

**Table 1:** Prevalence of malaria, HIV infection, and syphilis among pregnant women in the Ambanja district, Madagascar, from January 13, 2015, to September 20, 2019, at their first antenatal care visit (n=1443).

Variables		Participants, n (%)	95% CI
**HIV status**			
	Positive	18 (1.25)	0.7-1.96
	Negative	1290 (89.40)	87.7-90.9
	Invalid or not tested	135 (9.36)	7.9-11.0
**Syphilis status**			
	Positive	41 (2.84)	2.0-3.8
	Negative	1061 (73.53)	71.2-75.8
	Invalid or not tested	341 (23.63)	21.5-25.9
**Malaria status**			
	Positive	23 (1.59)	1.0-2.4
	Negative	813 (56.34)	53.7-58.9
	Invalid or not tested	607 (42.06)	39.5-44.7

### Timing of First Antenatal Care Visit

Table 2 shows that since the implementation of PANDA began in 2015, women tended to attend their first ANC visit earlier in their pregnancy. Comparing the timing of the first ANC visit in terms of pregnancy trimester by year, we observed a significant change from 2015 to 2019. In 2019, fewer women attended their first visit in the third trimester (57/277, 20.6%) than in 2015 (147/343, 42.9%). In addition, more women attended their first ANC visit in the second trimester in 2019 than in the previous years (*P<*.001).

**Table 2:** Timing of the first antenatal care visit during pregnancy by year among pregnant women in the Ambanja district, Madagascar.

Pregnancy trimester	Year, n (%)						*P* value
	2015 (n=341)	2016 (n=265)	2017 (n=197)	2018 (n=360)	2019 (n=276)		
First trimester	28 (8.2)	39 (14.7)	28 (14.2)	46 (12.8)	18 (6.5)	<.001	
Second trimester	168 (49.0)	148 (55.9)	131 (66.5)	237 (65.8)	201 (72.8)	N/A^a^	
Third trimester	147 (42.8)	78 (29.4)	38 (19.3)	77 (21.4)	57 (20.7)	N/A	

^a^N/A: not applicable.

### Mean Duration of Antenatal Care Visits Across Time and Associated Variables

In the univariate analysis, the mean visit duration decreased significantly by approximately 5 min from 2015 to 2019 and varied by approximately 10 min across visit orders (see the second table in Multimedia Appendix 1). There was no statistically significant difference in the mean duration of the visits by the place of residence (rural vs urban). The visit duration was significantly associated with the patient’s level of education, with longer visits for patients with less than primary education (mean 40.7 min; 95% CI 30.2-51.3 min) and patients who had completed university education (mean 25.3 min; 95% CI 24.4-26.3 min), compared with patients who had completed primary education (mean 23.3 min; 95% CI 22.9-23.8 min; *P*=.001) or secondary school (mean 22.8 min; 95% CI 22.0-23.4 min; *P*=.001). The visit duration also differed significantly by age, with significantly longer visits for women aged at least 35 years (mean 25.0 min; 95% CI 24.0-25.9 min) than for those aged between 16 and 20 years (22.9 min; 95% CI 22.3-23.5 min; *P*<.001). Patients who reported domestic violence during the visit had longer visit durations than those who did not report domestic violence (*P*<.001), and those who had running water at home had longer visit durations than those without running water at home. There were also significant differences in the visit duration by patient gravidity (longer duration with higher gravidity), parity (longer duration with higher parity), and trimester of pregnancy (shorter duration in the third [mean 21.7 min; 95% CI 21.3-22.1 min; *P*<.001] and second [mean 25.5 min; 95% CI 25.1-25.9 min; *P*<.001] trimesters, compared with the first trimester [mean 29.2 min; 95% CI 27.8-30.5 min]). The providers noted that the visit duration increased with parity and gravidity as they had to go through more detailed information about each past pregnancy and delivery. Active smokers had longer visit durations than did nonsmokers, and women with severe or moderate anemia had longer visit durations than women with no anemia or mild anemia. Finally, there were significant differences in the visit duration by HIV status (longer duration among patients with positive HIV status compared with those with negative or unknown status), syphilis status (longer duration among patients with unknown status compared with those with positive or negative status), and malaria status (longer duration among patients with positive status compared with those with negative or unknown status). In the multivariable model, the mean visit duration remained independently associated with the year, visit order, level of education, age category, domestic violence, availability of running water at home, and HIV and syphilis status (Multimedia Appendix 1).

**Figure 1:**
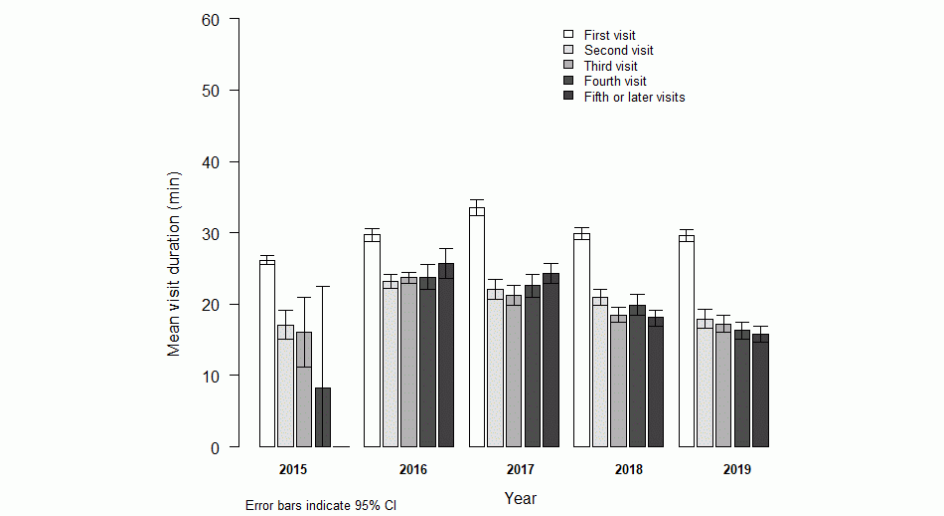
Mean antenatal care visit duration in minutes (95% CI) by year and visit order.

Figure 1 displays the mean visit duration by year and visit order. The duration of the first ANC visit remained stable over time. In 2015 and 2019, the mean duration decreased with each subsequent visit. In 2016 and 2017, the fifth visit was longer than the fourth visit, and the fourth visit was longer than the third visit in 2018.

All variables included in the multivariable model were significantly associated with the visit duration. These variables were the year, visit order, education, age, availability of running water at home, HIV status, and syphilis status.

### Number of Visits Per Woman by Year and Antenatal Coverage

**Figure 2:**
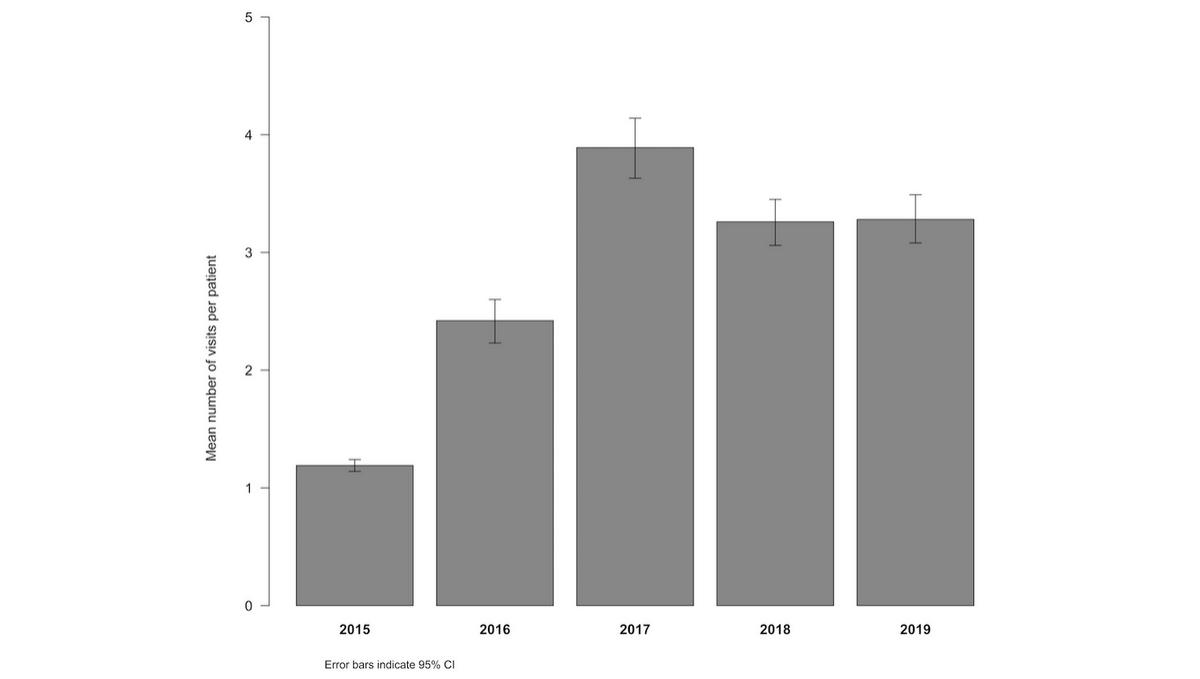
Mean number of antenatal care visits (95% CI) per patient by year (Kruskal-Wallis test; *P*<.001).

We observed a statistically significant increase in the number of visits per woman from 2015 to 2017. The number of visits per woman then became stable after the third year of implementing the PANDA system. Figure 2 illustrates the mean number of visits per woman per year.

## Discussion

### Principal Findings and Interpretation

This study has shown the potential of using an mHealth system to encourage providers to follow a standardized ANC visit and also contribute to increase women’s adherence and willingness to return for subsequent ANC visits in Ambanja, Madagascar.

According to the United Nations International Children's Emergency Fund data gathered in Madagascar in 2018, most women attended their first ANC visit in the third trimester of pregnancy, with only 45% attending their first visit in the second trimester or earlier [24]. In this study, we found that 73% of the women attended their first visit in the second trimester in 2019. This suggests that a systematically conducted ANC visit, supported and guided by mHealth technology, has the potential to boost women’s confidence in health care providers. Similarly, in a randomized controlled trial conducted in 2017 in Ethiopia, Atnafu et al [25] found that, after implementing an mHealth tool to assist health care providers in collecting and organizing patient data in medical records, about 60% of the patients attended their first ANC visit from the fourth to the sixth month of their pregnancies.

There is no recommended benchmark value for the duration of ANC visits as a quality indicator; however, we assumed that a provider would need at least 20 min for the first ANC visit (based on our clinical experience) to cover all the main topics that should routinely be part of a high-quality ANC visit. The duration of the first ANC visit, a quality indicator defined by the WHO, remained stable over the study period, indicating that the use of the mHealth device did not significantly lengthen the visit duration. We also found that the visit duration was positively associated with several patient risk factors. Low education, being over 35 years of age, experiencing domestic violence during pregnancy, having anemia, and having an HIV positive status were all associated with a longer ANC visit duration.

In terms of the completeness of the ANC visits, most pregnant women in our sample were tested for HIV during these visits (92.7%), compared with only 10% of pregnant women in an analysis of national-level data from 2018, which also reported that only 3% were receiving antiretroviral therapy to prevent vertical transmission [26]. Furthermore, HIV prevalence was 1.3% (95% CI 0.7-1.96%) in our sample, compared with 0.5% (95% CI 0.1-0.2%) in the 2018 national-level data. The prevalence of syphilis infection in our sample was 2.8% (95% CI 2.0-3.8%). Previous reports of the seroprevalence of syphilis in Madagascar ranged from 1.6% to 4.5% [27]. In addition, the national-level data indicated that only 68% of pregnant women in Madagascar had their blood pressure measured during ANC visits [24], compared with almost 100% of the women in our sample using the PANDA system.

In this study, we found that the number of ANC visits per pregnant woman tended to increase over the study period. In the study by Atnafu et al, [25] the number of ANC visits varied significantly by region, with 77% of pregnant women attending 1 to 4 visits and only 23% attending more than four ANC visits. It can be assumed that increasing women’s willingness to return for subsequent ANC visits may help prevent maternal morbidities that can put the mother and newborn at risk during pregnancy. Rosario et al [28] have shown that birth outcomes are directly related to ANC attendance (stillbirth: unadjusted odds ratio [OR]=0.34, 95% CI 0.16-0.70; abortion: OR=0.07, 95% CI 0.04-0.12) According to the national data, in 2018, only 51% of women in Madagascar attended at least four ANC visits [29].

### Strengths of the Study

This study analyzed data from a large population over a 5-year period following the implementation of the PANDA mHealth system to support good quality ANC in a resource-constrained setting (Madagascar). The indicators in our study, such as the timing of the first ANC visit, the visit duration, and the number of visits, strongly suggest that this mHealth system encourages the performance of a standardized ANC visit and thereby facilitates the provision of high-quality ANC services. This study has shown the benefits of the PANDA mHealth system for both providers and patients. The providers received guidance on how to conduct standardized ANC of good quality, and they also received a record of all important patient data that can be used in follow-up visits. The women and their families received ongoing education and encouragement to seek appropriate care throughout the ANC and postpartum visits conducted with the PANDA system. The system improved communication between the health care workers and patients, facilitated continuous education, and encouraged the patients to play a more active role in the decision-making processes related to their health.

### Limitations of the Study

Our study has several limitations. The most significant limitation is the lack of a control group to compare with participants using the PANDA mHealth system. Instead, we used national-level surveys as a standard reference—the 2009 Madagascar Demographic and Health Survey and the 2018 Multiple Indicator Cluster Survey. Although our results differed significantly from the findings of these two national-level surveys, the lack of a comparison group and a randomized design limits the conclusiveness of our findings. D-tree International’s study on safer deliveries in Tanzania, which also lacked a control group, demonstrated that the examined mHealth program was a success as it reached over 13,000 pregnant women in Zanzibar. The implementation of this mHealth system was described as a success as it was linked to unprecedented rates of both service delivery and postpartum attendance, even though this mHealth system did not provide support for either ANC or postpartum visit content or quality [30]. A second limitation is that we did not measure health outcomes to evaluate the effectiveness of the mHealth intervention; we only began to collect data on postpartum visits and delivery outcomes in the last year of the study. However, the study by Lund et al [31] in Tanzania showed a trend toward improved time and quality after the implementation of a mobile phone intervention for patients in a cluster randomized control study in Tanzania. They found that the mobile phone intervention was associated with not only an increase in ANC attendance, 44% of the women in the intervention group received four or more ANC visits versus 31% in the control group (R, 2.39; 95% CI 1.03-5.55), but also a trend toward improved timing and quality of ANC services, although the difference was not significant. We also did not explore the cost effectiveness or durability of the PANDA system, compared with the existing paper-based checklist. These aspects need to be explored in future studies. Finally, we trained 13 providers to use the PANDA mHealth system, and we did not explore their satisfaction with the system. The providers were not selected randomly; rather, an opportunistic selection was employed.

Several questions about the use and diffusion of the PANDA mHealth system remain open. These questions relate to, for example, dealing with the low quality of service delivery and the scalable and sustainable integration of the PANDA system in the local health system.

### Conclusions

This study shows that the PANDA mHealth system has the potential to improve the quality of ANC in a resource-limited setting, modify the behavior of providers by providing standardized ANC visits, and increase patient compliance. Mobile technology should not be considered a stand-alone health intervention for ANC; rather, it is a strategic tool for improving the delivery and quality of maternal health care. Further studies are necessary to better understand the conditions under which behavioral changes occur and persist over time with the use of mHealth systems such as PANDA as well as whether undesired behavioral changes may also arise with the use of mHealth in ANC settings.

## Appendix

Multimedia Appendix 1 Sociodemographic and clinical characteristics of pregnant women in the Ambanja district, Madagascar, from January 13, 2015, to September 20, 2019, at their first antenatal care visit (Table 1). Sociodemographic and clinical variables that were significantly associated with antenatal care visit duration among pregnant women in the Ambanja district, Madagascar (univariate and multivariable analyses) (Table 4).

## Abbreviations

ANC: antenatal care
LMIC: low- and middle-income country
mHealth: mobile health
OR: odds ratio
PANDA: Pregnancy and Newborn Diagnostic Assessment
WHO: World Health Organization
