# Supplementary material for: Improving the Quality of Antenatal Care Using Mobile Health in Madagascar: Five-Year Cross-Sectional Study
Source: JMIR Mhealth Uhealth. 2020 Jul 8;8(7):e18543. doi: 10.2196/18543 (PMC7381010; doi:10.2196/18543)
Supplement: Multimedia Appendix 1 [file mhealth_v8i7e18543_app1.docx]

**Table 1.** Sociodemographic and clinical characteristics of pregnant women in the Ambanja district, Madagascar, from January 13, 2015, to September 20, 2019, at their first antenatal care visit.

| Variable | | Values |
| --- | --- | --- |
| **Age (years; n=1442)** | | |
|  | Mean (SD) | 24.4 (6.8) |
|  | Median | 23 |
| **Marital status, n (%; n=1441)** | | |
|  | Married | 1185 (82.23) |
|  | Single | 224 (15.54) |
|  | Living with a family member | 32 (2.22) |
| **Highest level of education, n (%; n=1441)** | | |
|  | Primary school | 819 (56.83) |
|  | Secondary school | 404 (28.04) |
|  | University | 115 (7.98) |
|  | Less than primary school | 3 (0.21) |
|  | Other or unknown | 102 (7.08) |
| Illiteracy, n (%; n=1438) | | 96 (6.68) |
| No running water at home, n (%; n=1441) | | 1337 (92.78) |
| Urban residency, n (%; n=1443) | | 1166 (80.80) |
| **Professional category, n (%; n=1443)** | | |
|  | Housewife | 846 (58.63) |
|  | Field worker | 237 (16.42) |
|  | Student | 36 (2.49) |
|  | Other or unknown | 324 (22.45) |
| Domestic violence, n (%; n=1438) | | 67 (4.66) |
| **Gravidity, n (%; n=1443)** | | |
|  | 1 | 479 (33.19) |
|  | 2 | 416 (28.83) |
|  | 3 | 256 (17.74) |
|  | 4 | 121 (8.38) |
|  | 5 | 73 (5.06) |
|  | 6 | 48 (3.33) |
|  | ≥7 | 50 (3.46) |
| **Parity, n (% N=1443)** | | |
|  | 0 | 574 (39.78) |
|  | 1 | 392 (27.16) |
|  | 2 | 233 (16.15) |
|  | ≥3 | 244 (16.91) |
| **Pregnancy trimester, n (%; n=1441)** | | |
|  | First | 159 (11.03) |
|  | Second | 885 (61.42) |
|  | Third | 397 (27.55) |
| Active smoking, n (%; n=1436) | | 27 (1.88) |
| Previous CS, n (%n=1441) | | 118 (8.19) |
| History of postpartum hemorrhage, n (%; n=1441) | | 42 (2.91) |
| Two or more stillbirths, n (%; n=1441) | | 5 (0.35) |
| Previous home delivery, n (%; n=1441) | | 307 (21.30) |
| Alcohol consumption, n (%; n=1436) | | 8 (0.56) |
| Past tuberculosis, n (%; n=1436) | | 15 (1.04) |
| Tetanus immunization, n (%; n=985) | | 154 (15.63) |
| Fetal movement, n (%; n=1408) | | 1094 (77.70) |
| Fetal movement during the past 12 hours, n (%; n=1121) | | 1069 (95.36) |
| **SBP^a^ (****mm Hg; n=1424)** | | |
|  | Mean (SD) | 107.7 (11.8) |
|  | Median | 107 |
| **DBP^b^ (mm Hg; n=1424)** | | |
|  | Mean (SD) | 66.6 (10.3) |
|  | Median | 66 |
| High blood pressure (defined as SBP≥130 mm Hg or DBP≥80 mm Hg), n (%;1424) | | 160 (11.24) |
| Severe high blood pressure (defined as SBP≥140 mm Hg or DBP≥90 mm Hg), n (%; N=1406)) | | 45 (3.20) |
| **Heart rate (beats per minute; n=1424)** | | |
|  | Mean (SD) | 92.2 (12.2) |
|  | Median | 92 |
| **Glucose (mg/dL; n=1155)** | | |
|  | Mean (SD) | 74.2 (22.9) |
|  | Median | 77 |
| Pregnancy with diabetes (defined as glucose≥126 mg/dL), n (%; n=1133) | | 17 (1.50) |
| **Hemoglobin (g/dL; n=1275)** | | |
|  | Mean (SD) | 10.3 (1.3) |
|  | Median | 10.3 |
| **Anemia, n (%; n=1275)** | | |
|  | Anemic (defined as hemoglobin <11 g/dL) | 878 (68.86) |
|  | Mild anemia (defined as hemoglobin 10 g/dL-11 g/dL) | 421 (33.02) |
|  | Moderate anemia (defined as hemoglobin 7 g/dL-9.9 g/dL) | 450 (35.30) |
|  | Severe anemia (defined as hemoglobin <7 g/dL) | 7 (0.55) |
| **Received treatment for anemia, n (%; n=1440)** | | |
|  | No | 896 (62.22) |
|  | Yes | 538 (37.36) |
|  | Unknown | 6 (0.42) |
| **Received treatment for worms, n (%; n=1440)** | | |
|  | No | 955 (66.32) |
|  | Yes | 477 (33.12) |
|  | Unknown | 8 (0.55) |
| **Planned pregnancy (in 2018 and 2019 only), n (%; n=529)** | | |
|  | Unplanned | 191 (36.10) |
|  | Planned | 338 (63.89) |
| **Contraceptive use before pregnancy (in 2018 and 2019 only), n (%; n=329)** | |  |
|  | Long-acting progesterone | 170 (51.67) |
|  | Oral contraception | 36 (10.94) |
|  | Intrauterine device | 15 (4.56) |
|  | Condom | 1 (0.30) |
|  | Traditional methods | 106 (32.21) |
|  | Other | 1 (0.30) |
| **Contraceptive use after pregnancy (in 2018 and 2019 only), n (%; n=493)** | | |
|  | No | 20 (4.06) |
|  | Yes | 473 (95.94) |

^a^SBP: systolic blood pressure.

^b^DBP: diastolic blood pressure.

**Table 4**. Sociodemographic and clinical variables that were significantly associated with antenatal care visit duration among pregnant women in the Ambanja district, Madagascar (univariate and multivariable analyses).

| Variable | | Univariate analysis | | Multivariable analysis | |
| --- | --- | --- | --- | --- | --- |
|  | | Estimated mean duration (95% CI)^a^ | *P* value^b^ | Estimated mean duration (95% CI)^a^ | *P* value^b^ |
|  | |  |  |  |  |
| **Year** | | —^c^ | <.001 | — | <.001 |
|  | 2016 | 26.2 (25.5-26.9) | — | 25.7 (25.0-26.3) | — |
|  | 2017 | 25.3 (24.7-26.0) | .097 | 25.6 (25.0-26.2) | .92 |
|  | 2018 | 22.7 (22.2-23.2) | <.001 | 22.6 (22.1-23.1) | <.001 |
|  | 2019 | 20.5 (19.9-21.1) | <.001 | 20.8 (20.3-21.3) | <.001 |
| **Visit order** | | — | <.001 | — | <.001 |
|  | First visit | 30.4 (30.0-30.9) | — | 27.7 (26.9-28.4) | — |
|  | Second visit | 20.9 (20.3-21.4) | <.001 | 21.7 (21.1-22.2) | <.001 |
|  | Third visit | 19.6 (19.0-20.2) | <.001 | 21.0 (20.3-21.7) | <.001 |
|  | Fourth visit | 19.9 (19.2-20.6) | <.001 | 21.4 (20.6-22.1) | <.001 |
|  | ≥Fifth visit | 19.1 (18.4-19.9) | <.001 | 21.0 (20.3-21.8) | <.001 |
| **Residence** | | — | .09 | — | — |
|  | Rural | 22.4 (20.7-24.1) | — | — | — |
|  | Urban | 23.5 (23.1-23.8) | — | — | — |
| **Education** | | — | <.001 | — | <.001 |
|  | Primary school | 23.3 (22.9-23.8) | — | 23.1 (22.8-23.5) | — |
|  | Secondary school | 22.8 (22.2-23.4) | .27 | 23.0 (22.5-23.5) | .73 |
|  | University | 25.3 (24.4-26.3) | <.001 | 25.1 (24.3-25.9) | <.001 |
|  | Less than primary school | 40.7 (30.2-51.3) | .001 | 35.0 (26.4-43.6) | .007 |
|  | Other or unknown | 22.7 (21.1-24.3) | .97 | 22.8 (21.4-24.1) | .595 |
| **Age group (years)** | | — | .001 | — | <.001 |
|  | 16-20 | 22.9 (22.3-23.5) | — | 22.6 (22.1-23.1) | — |
|  | <16 | 22.7 (21.1-24.2) | .77 | 21.8 (20.4-23.3) | .34 |
|  | 21-34 | 23.7 (23.3-24.1) | .03 | 23.5 (23.1-23.8) | .006 |
|  | ≥35 | 25.0 (24.0-25.9) | <.001 | 24.9 (24.1-25.7) | <.001 |
| **Domestic violence during pregnancy** | | — | <.001 | — | — |
|  | No | 23.5 (23.1-23.8) | — | — | — |
|  | Yes | 26.7 (25.0-28.4) |  | — | — |
| **Running water at home** | | — | <.001 | — | .005 |
|  | No | 23.4 (23.1-23.7) | — | 23.3 (23.0-23.5) | — |
|  | Yes | 27.1 (25.7-28.5) | — | 25.5 (24.0-27.1) | — |
| **Gravidity** | | — | <.001 | — | — |
|  | 1 | 22.7 (22.2-23.2) | — | — | — |
|  | 2 | 23.6 (23.1-24.2) | .02 | — | — |
|  | 3 | 23.9 (23.2-24.6) | .007 | — | — |
|  | 4 | 24.1 (23.0-25.2) | .02 | — | — |
|  | 5 | 24.6 (23.2-26.0) | .02 | — | — |
|  | 6 | 24.1 (22.4-25.9) | .12 | — | — |
|  | ≥7 | 26.1 (24.3-27.9) | <.001 | — | — |
| **Parity** | | — | .0012 | — | — |
|  | 0 | 23.0 (22.5-23.5) | — | — | — |
|  | 1 | 23.5 (22.9-24.1) | .19 | — | — |
|  | 2 | 23.9 (23.2-24.7) | .04 | — | — |
|  | ≥3 | 24.8 (24.0-25.6) | <.001 | — | — |
| **Pregnancy trimester** | | — | <.001 | — | — |
|  | First | 29.2 (27.8-30.5) | — | — | — |
|  | Second | 25.5 (25.1-25.9) | <.001 | — | — |
|  | Third | 21.7 (21.3-22.1) | <.001 | — | — |
| **Active smoking** | | — | .001 | — | — |
|  | No | 23.5 (23.2-23.8) | — | — | — |
|  | Yes | 29.0 (25.8-32.3) | — | — | — |
| **Alcohol consumption** | | — | .399 | — | — |
|  | No | 23.5 (23.2-23.9) | — | — | — |
|  | Yes | 26.0 (20.3-31.8) | — | — | — |
| **High blood pressure (systolic blood pressure≥130 mm Hg or diastolic blood pressure≥80 mm Hg)** | | — | .07 | — | — |
|  | No | 23.5 (23.2-23.8) | — | — | — |
|  | Yes | 24.5 (23.5-25.5) | — | — | — |
| **Severity of anemia** | | — | <.001 | — | — |
|  | Not anemic | 24.6 (23.9-25.2) | — | — | — |
|  | Mild anemia | 23.1 (22.6-23.7) | .001 | — | — |
|  | Moderate anemia | 24.8 (24.2-25.4) | .56 | — | — |
|  | Severe anemia | 27.9 (22.9-33.0) | .19 | — | — |
| **Anemia** | | — | .09 | — | — |
|  | No | 24.6 (23.9-25.2) | — | — | — |
|  | Yes | 23.9 (23.5-24.3) | — | — | — |
| **HIV status** | | — | <.001 | — | <.001 |
|  | Negative | 29.3 (28.8-29.7) | — | 24.9 (24.0-25.8) | — |
|  | Positive | 30.3 (26.8-33.8) | .5649 | 28.1 (24.4-31.8) | .09 |
|  | Invalid or not tested | 20.0 (19.6-20.3) | <.001 | 22.6 (22.1-23.1) | <.001 |
| **Syphilis status** | | — | <.001 | — | <.001 |
|  | Negative | 29.2 (28.7-29.7) | — | 25.5 (24.6-26.4) | — |
|  | Positive | 28.5 (26.2-30.7) | .51 | 25.3 (22.8-27.9) | .89 |
|  | Invalid or not tested | 20.8 (20.5-21.2) | <.001 | 22.6 (22.2-23.0) | <.001 |
| **Malaria status** | | — | <.001 | — | — |
|  | Negative | 29.5 (28.9-30.1) | — | — | — |
|  | Positive | 28.5 (25.2-31.8) | .56 | — | — |
|  | Invalid or not tested | 21.6 (21.2-21.9) | <.001 | — | — |

^a^Obtained from a mixed linear model with the patient as a random variable.

^b^P-values are provided for the comparisons between each category to the first one (reference).
